# Einsamkeit bei Arbeitslosen mit psychischen Erkrankungen

**DOI:** 10.1007/s00103-024-03933-2

**Published:** 2024-08-07

**Authors:** Felix S. Hussenoeder, Maria Koschig, Ines Conrad, Alexander Pabst, Katharina Gatzsche, Luise Bieler, Mathias Alberti, Katarina Stengler, Steffi G. Riedel-Heller

**Affiliations:** 1 Institut für Sozialmedizin, Arbeitsmedizin und Public Health, Medizinische Fakultät, Universität Leipzig, Philipp-Rosenthal-Str. 55, 04103 Leipzig, Deutschland; 2 Zentrum für Seelische Gesundheit, HELIOS Park-Klinikum Leipzig, Leipzig, Deutschland

**Keywords:** Psychische Gesundheit, Soziales Netzwerk, Depression, Angst, Somatisierung, Mental health, Social network, Depression, Anxiety, Somatization

## Abstract

**Hintergrund:**

Einsamkeit ist ein weitverbreitetes Phänomen und steht in Zusammenhang mit gesundheitlichen Beeinträchtigungen. Dabei stellen Arbeitslose mit psychischen Erkrankungen (ALPE) eine Hochrisikogruppe in Hinblick auf Gesundheit und berufliche Wiedereingliederung dar. Ziel dieser Studie ist ein besseres Verständnis der Zusammenhänge zwischen Soziodemografie, psychischer Gesundheit und Einsamkeit bei ALPE.

**Methoden:**

Für die vorliegende Studie wurden die Fragebögen von 526 arbeitslosen Personen im ALG-2-Bezug und mit mindestens einer psychiatrischen Diagnose aus dem Erhebungszeitraum 09/2020–09/2023 ausgewertet. Es wurden 2 Regressionsanalysen mit robusten Schätzern und der Outcome-Variable Einsamkeit (University of California, Los Angeles, Loneliness Scale, UCLA) durchgeführt. Bei der ersten Regression wurde der alleinige Prädiktor soziales Netzwerk (Lubben Social Network Scale, LSNS-6) verwendet, bei der zweiten wurden Schulden (Ja/Nein), Depression (Patient Health Questionnaire, PHQ-9), Angst (Mini-Symptom-Checkliste, MSCL) und Somatisierung (MSCL) ergänzt sowie Alter, Geschlecht, Bildung und Zusammenleben mit Partner als Kontrollvariablen.

**Ergebnisse:**

Es zeigen sich ein signifikant negativer Zusammenhang zwischen sozialem Netzwerk und Einsamkeit sowie ein signifikant positiver Zusammenhang zwischen hoher Bildung, Depression, Angst und Einsamkeit.

**Diskussion:**

Bei den ALPE fanden sich im Vergleich zu anderen Studien hohe Belastungen durch Depression, Angst, Somatisierung und Einsamkeit. Die identifizierten Zusammenhänge zwischen sozialem Netzwerk, psychischer Gesundheit und Einsamkeit verdeutlichen die Wichtigkeit von psychologischen Screeningverfahren und/oder Diagnostik in dieser Hochrisikogruppe und bieten Ansatzpunkte für die Prävention.

## Abstract (en)

**Background:**

Loneliness is a widespread phenomenon, and it is associated with a variety of health problems and diseases. Unemployed individuals diagnosed with a mental illness (UMIs) are at a high risk of experiencing loneliness, with serious repercussions for their health and vocational rehabilitation. With this study we wanted to better understand the associations between sociodemographic variables, mental health, and loneliness in UMIs.

**Methods:**

We analyzed the data of 526 unemployed individuals that received means-tested benefits as well as at least one psychiatric diagnosis. Data were collected between September 2020 and September 2023. We conducted two robust regressions with loneliness (University of California, Los Angeles, Loneliness Scale, UCLA) as an outcome (*N* = 526). In the first regression, we used social network as a predictor (Lubben Social Network Scale, LSNS-6); in the second regression we included the additional predictors personal debt (yes/no), depression (Patient Health Questionnaire, PHQ-9), anxiety (Mini-Symptom-Checklist,MSCL), and somatization (MSCL) as well as age, gender, education, and living with a partner as control variables.

**Results:**

We found a significant negative association between social network and loneliness, and a significant positive association between a high level of education, depression, anxiety, and loneliness.

**Conclusions:**

Unemployed individuals diagnosed with a mental illness in our sample exhibited high levels of depression, anxiety, somatization, and loneliness. The associations between social network, mental health, and loneliness that we found emphasize the importance of psychological screening and/or diagnostics, and they highlight areas for prevention.

## Hintergrund

Einsamkeit ist ein weitverbreitetes Phänomen [1] und steht oftmals in Zusammenhang mit einer beeinträchtigten Gesundheit. So zeigen Studien die Verbindung zwischen Einsamkeit und Herzerkrankungen [2], kognitivem Abbau im Alter [3], Depression [4], Substanzmissbrauch [5] sowie Essstörungen [6]. Auch die in der Literatur diskutierten potenziellen Ursachen von Einsamkeit sind zahlreich und divers; hier finden sich beispielsweise Schüchternheit [7], Stress [8] und zwanghafte Internetnutzung [9]. Ähnlich sieht es bei den in der Literatur identifizierten Risikogruppen aus, unter denen sich z. B. Personen befinden, welche einer ethnischen Minderheit angehören, sowie Alleinlebende, Arbeitslose, psychisch Kranke, junge Frauen oder Adoleszente mit einem niedrigen Bildungsniveau [10]. Es ist naheliegend, dass in unterschiedlichen Risikogruppen auch unterschiedliche Faktoren(kombinationen) mit der Entstehung und Aufrechterhaltung der Einsamkeit in Verbindung stehen. Um Bedarfe besser zu identifizieren und wirkungsvolle Präventionsmaßnahmen zu entwickeln, ist es dementsprechend wichtig, spezifische Risikogruppen in den Fokus zu nehmen und diese gezielt zu adressieren. In unserer Studie konzentrieren wir uns deshalb auf eine Hochrisikogruppe: arbeitslose Personen mit psychischen Erkrankungen (ALPE).

ALPE sind nicht nur doppelt belastet, die Forschung zeigt auch, dass sich Arbeitslosigkeit und eine reduzierte psychische Gesundheit gegenseitig verstärken können, sodass ein sog. Teufelskreis entsteht [11–13], bei dem einerseits eine psychische Belastung/Erkrankung die Aufnahme oder Weiterführung einer Arbeit verhindert und andererseits die (länger andauernde) Arbeitslosigkeit die Auftretenswahrscheinlichkeit und Schwere einer psychischen Erkrankung erhöht. Zugrunde liegende Mechanismen können hierbei beispielsweise der Wegfall von ökonomischen Ressourcen, sozialen Beziehungen und sinnstiftenden Tätigkeiten durch die Arbeitslosigkeit einerseits sowie die Stigmatisierung von Menschen mit psychischen Erkrankungen am Arbeitsmarkt andererseits sein. Darüber hinaus zeigt die Forschung, dass Wechselwirkungen auch zwischen Einsamkeit und Arbeitslosigkeit [14] und zwischen Einsamkeit und psychischer Gesundheit existieren [15], welche zu einer gegenseitigen Stabilisierung und Verstärkung beitragen können. Damit spielt Einsamkeit für die Gesundheit und die berufliche Wiedereingliederung von ALPE eine zentrale Rolle. Zudem sind arbeitslose Personen häufiger von Schulden betroffen, wie die Ergebnisse des European Household Finance and Consumption Survey zeigen [16], und es existieren Hinweise darauf, dass Schulden Einsamkeit verstärken können [17].

Ein tieferes, wissenschaftsbasiertes Verständnis der Zusammenhänge zwischen psychischer Gesundheit und Einsamkeit bei ALPE kann dazu beitragen, nicht nur Verbesserungen für Betroffene zu erreichen, sondern auch Entlastungen für das Sozialsystem. Dementsprechend interessiert uns, welche Rolle Einsamkeit bei ALPE spielt und welche Faktoren damit in Zusammenhang stehen. Wir gehen davon aus, dass das soziale Netzwerk eine Rolle spielt, d. h., dass das objektive Ausmaß der sozialen Eingebundenheit im Zusammenhang mit der subjektiv erlebten Einsamkeit steht, aber auch soziodemografische Faktoren. Des Weiteren interessieren uns die Wechselwirkungen zwischen dem Vorliegen von Schulden sowie der psychischen Gesundheit und Einsamkeit.

## Methoden

### Studiendesign

Bei dem Projekt „LIPSY: Leipzig – Individual Placement and Support für psychisch kranke Menschen“ geht es darum, die Erwerbsfähigkeit von arbeitslosen Menschen im ALG-2-Bezug mit psychischen Erkrankungen zu erhalten oder wiederherzustellen und ihnen eine nachhaltige Arbeitsmarktintegration zu ermöglichen. Hierzu erhalten Kunden am Jobcenter die Möglichkeit, einen Screeningbogen auszufüllen, um potenzielle psychische Belastungen zu identifizieren. Personen mit Belastungen werden dann zu einer umfangreichen psychiatrischen Diagnose weitergeleitet. Im Projektverlauf erhalten die Studienteilnehmer*innen, in Abhängigkeit vom Schweregrad der psychischen Erkrankung, Beratung hinsichtlich Behandlungsmöglichkeiten und eine Weiterleitung ins Versorgungssystem. Einige der Teilnehmer*innen können im Rahmen des Projektes an einem Trial zur Erprobung eines Ansatzes der „unterstützten Beschäftigung“ (Supported Employment) partizipieren [18]. Die begleitende Evaluation wird mittels Selbstbeurteilungsfragebögen zu 4 Zeitpunkten durchgeführt. In der ersten Befragung (Baseline t0) wird mit den Teilnehmer*innen eine umfassende psychiatrische Diagnostik durchgeführt. Für die vorliegende Studie wurden die ersten 600 Fragebögen (t0) aus dem Erhebungszeitraum 09/2020–09/2023 berücksichtigt, nach der Bereinigung des Datensatzes und der Entfernung von Fragebögen mit fehlenden Werten beinhaltete das Analysesample 526 Personen. Alle Teilnehmer*innen befanden sich im ALG-2-Bezug und wiesen mindestens eine psychiatrische Diagnose auf (ICD-Code F).

### Instrumente

*Soziodemografische Variablen.* Die Teilnehmer*innen machten Angaben zu Alter, Geschlecht, Bildung (Schulabschluss), Zusammenleben mit dem/r Partner/in und zum Vorliegen von Schulden. Die Angaben zur Bildung wurden in 4 Kategorien unterteilt: (1) ohne Hauptschulabschluss, (2) niedrig (Hauptschulabschluss), (3) mittel (Realschulabschluss oder Abschluss der Polytechnischen Oberschule 10. Klasse), (4) hoch (Fachhochschulreife, Abschluss Fachoberschule, Allgemeine/fachgebundene Hochschulreife/Abitur).

*Soziales Netzwerk. *Mithilfe der kurzen Version der Lubben Social Network Scale wurde die Größe des sozialen Netzwerks bei den Teilnehmer*innen abgebildet (LSNS‑6; [19]). Die Skala beinhaltet 6 Items, welche auf einer 6‑stufigen Skala beantwortet werden können und die soziale Vernetzung in Bezug auf Familienangehörige sowie Freunde und Nachbarn erfassen. Theoretisch bewegen sich die Werte zwischen 0 und 30, wobei höhere Werte einem größeren Netzwerk entsprechen. Soziale Isolation liegt bei Werten <12 vor [19].

*Depressivität.* Der Patient Health Questionnaire‑9 (PHQ‑9, [20, 21]) wurde genutzt, um die Depressivität der Teilnehmenden zu messen. Der Test beinhaltet 9 symptomorientierte Items, welche auf einer Likert-Skala von 0 = „überhaupt nicht“ bis 3 = „beinahe jeden Tag“ bewertet werden können, wobei höhere Werte einer höheren Depressivität entsprechen.

*Angst und Somatisierung. *Die „Mini-Symptom-Checkliste“ wurde verwendet, um das Ausmaß von Angst und Somatisierung mit jeweils 6 Items zu erfassen, welche auf einer Skala von 0 = „überhaupt nicht“ bis 4 = „sehr stark“ beantwortet werden können [22]. Die Werte für beide Variablen können theoretisch zwischen 0 und 24 liegen.

*Einsamkeit.* Einsamkeit wurde mit der deutschen 3‑Item-SOEP-Version der UCLA Loneliness Scale gemessen [23, 24]. Die Items können auf einer Skala von 1 = „nie/selten“ bis 3 = „oft“ bewertet werden. Damit liegen die Ergebnisse für Einsamkeit zwischen 3 und 9.

### Statistische Analyse

Für die statistischen Analysen nutzten wir SPSS Version 27.0 (IBM; Armonk, NY, USA). Es wurden 2 Regressionsanalysen durchgeführt. Bei der ersten bildete das soziale Netzwerk den alleinigen Prädiktor und Einsamkeit den Outcome. Bei der zweiten Analyse waren soziales Netzwerk, Schulden, Depressivität, Angst und Somatisierung die Prädiktoren und Einsamkeit die Outcomevariable. Alter, Geschlecht, Bildung und Zusammenleben wurden als Kontrollvariablen hinzugefügt. Die Analysen wurden mit robusten Standardfehlern durchgeführt [25].

## Ergebnisse

Unsere Stichprobe enthielt 526 Personen mit einem Durchschnittsalter von 35,7 Jahren (Range: 18–63 Jahre), 51,3 % waren weiblich. 61,0 % der Teilnehmer*innen waren sozial isoliert (LSNS-6 < 12). Insgesamt hatten 10,3 % der Befragten keinen Hauptschulabschluss, 47,5 % keinen beruflichen Abschluss und 51,9 % gaben an, verschuldet zu sein. Tab. 1 gibt einen Überblick über die Eigenschaften der untersuchten Stichprobe.

**Tab. 1:** Allgemeine Merkmale der Stichprobe

Merkmale	Stichprobe (*N* = 526)
*Alter (Jahre)*	35,7 (11,1)
*Geschlecht („weiblich“)*	270 (51,3 %)
*Schulabschluss*^*1*^	
Ohne Hauptschulabschluss	54 (10,3 %)
Niedrig (Hauptschule)	123 (23,4 %)
Mittel (Realschule, Polytechnische Oberschule)	214 (40,7 %)
Hoch (Abschluss Fachoberschule, Hochschulreife)	135 (25,7 %)
*Kein beruflicher Ausbildungsabschluss (und nicht in Ausbildung)*	250 (47,5 %)
*Zusammenleben mit Partner („Ja“)*	94 (17,9 %)
*Schulden („Ja“)*	273 (51,9 %)
*Soziales Netzwerk (LSNS‑6; Range: 0–30)*	10,1 (5,7)
*Soziale Isolation (LSNS‑6* *<* *12)*^*1*^	321 (61,0 %)
*Depression (PHQ‑9; Range: 0–27)*	13,8 (5,4)
*Angst (MSCL; Range: 0–24)*	7,3 (5,1)
*Somatisierung (MSCL; Range: 0–24)*	5,6 (4,7)
*Einsamkeit (UCLA; Range: 3–9)*	5,9 (1,9)

*LSNS* Lubben Social Network Scale, *PHQ* Patient Health Questionnaire, *MSCL* Mini-Symptom-Checkliste, *UCLA* University of California, Los Angeles

Bei kontinuierlichen Variablen werden der Mittelwert und die Standardabweichung (in Klammern), bei kategorischen Variablen werden Zahlen und Prozentwerte (in Klammern) angegeben

^1^Definition nach [38]

Tab. 2 zeigt einen signifikanten Zusammenhang zwischen einem größeren sozialen Netzwerk und weniger Einsamkeit, sowohl in der einfachen als auch in der multiplen Regression. Zudem zeigt sich ein signifikant positiver Zusammenhang zwischen hoher Bildung, Depression, Angst und Einsamkeit. Es existierte kein signifikanter Zusammenhang zwischen Schulden, Somatisierung und Einsamkeit.

**Tab. 2:** Vorhersage von Einsamkeit durch das soziale Netzwerk ohne und mit zusätzlichen Kontrollvariablen und Prädiktoren (standardisierte Regressionskoeffizienten; *N* = 526)

Merkmale	Einsamkeit	
	β	β
*Soziales Netzwerk*	−0,14***	−0,13**
*Alter (Jahre)*	–	−0,00
*Geschlecht (Ref. „weiblich“)*	–	−0,01
*Schulabschluss (Ref. „ohne Abschluss“)*		
Niedrig	–	−0,03
Mittel	–	0,03
Hoch	–	0,15*
*Zusammenleben mit Partner (Ref. „Nein“)*	–	0,00
*Schulden (Ref. „Nein“)*	–	−0,02
*Depression*	–	0,23***
*Angst*	–	0,17**
*Somatisierung*	–	−0,10
R^2^	0,02	0,14

*Ref*. Referenzkategorie

*p ≤ 0,05; **p ≤ 0,01; ***p ≤ 0,001

## Diskussion

Unsere Ergebnisse zeigen eine hohe Belastung der Stichprobe durch Depression, Angst, Somatisierung und Einsamkeit, einen negativen Zusammenhang zwischen sozialem Netzwerk und Einsamkeit sowie einen positiven Zusammenhang zwischen Depression, Angst und Einsamkeit. Das heißt, dass ein kleines soziales Netzwerk sowie erhöhte Werte von Depression und Angst mit hoher Einsamkeit einhergehen.

Die geringere Einsamkeit bei Personen mit größeren Netzwerken findet sich auch in anderen Studien mit sehr unterschiedlichen Populationen, beispielsweise bei älteren Erwachsenen in Spanien [26], Personen mit einer Borderline-Persönlichkeitsstörung [27] oder Befragungsteilnehmer*innen während der COVID-19-Pandemie [28]. Während die ALPE in unserer Befragung einen durchschnittlichen Netzwerkgrößenscore von 10,1 aufweisen, zeigt eine aktuelle Studie – welche ebenfalls in Leipzig durchgeführt wurde – einen Durchschnittswert von 17,6 bei der Allgemeinbevölkerung [29]. Auch im Vergleich zu den psychisch erkrankten, älteren Personen einer walisischen Studie (15,9) weisen die Teilnehmer*innen unserer Studie deutlich niedrigere Werte auf [30]. Zudem weist die Studie von Röhr et al. [29] eine Häufigkeit von 14,8 % für soziale Isolation bei arbeitslosen Personen aus, während diese in unserer Stichprobe bei 61,0 % liegt. Auch wenn man davon ausgehen kann, dass unsere Studienteilnehmer*innen im Durchschnitt länger von Arbeitslosigkeit betroffen waren, als dies bei Röhr et al. der Fall war, und strengere Kriterien hinsichtlich der Diagnose einer psychischen Erkrankung erfüllen mussten als bei Evans et al., so legen unsere Ergebnisse dennoch nahe, dass ALPE noch einmal deutlich weniger sozial vernetzt sind als Personen, die „nur“ arbeitslos oder „nur“ psychisch krank sind.

Ausgehend von der zentralen Bedeutung sozialer Netzwerke für das Empfinden von Einsamkeit und dem Fakt, dass die Public-Health-Bedeutung von Einsamkeit in Deutschland bisher weitgehend unterschätzt wurde [31], sollte hier ein Schwerpunkt auf der Prävention liegen. Diese könnte sich auf den Ausbau und die Förderung von sozialen Beziehungen und Interaktionen im direkten Umfeld konzentrieren, um der sozialen Isolation und den negativen Verstärkungsprozessen zwischen sozialer Isolation, psychischer Erkrankung und Arbeitslosigkeit wirkungsvoll entgegenzutreten. Interventionen könnten beispielsweise das „Verschreiben“ sozialer Aktivitäten beinhalten [32] und sich gut in das ohnehin bereits existierende System von Fördermaßnahmen für Personen ohne Arbeit integrieren lassen.

Anders als beispielsweise im polnischen Household Panel [17] ergab sich in unserer Befragung kein signifikanter Zusammenhang zwischen Schulden und Einsamkeit. Unsere Ergebnisse sind damit auf einer Linie mit einer aktuellen niederländischen Studie [33], die zwar einen positiven Zusammenhang zwischen subjektiver Belastung durch Schulden und sozialer Einsamkeit belegt, diesen jedoch nicht für die objektive Belastung nachweist, welche ja auch in unserer Studie erhoben wurde. Unabhängig davon ist festzuhalten, dass mehr als die Hälfte der befragten ALPE angab, verschuldet zu sein, und dies einen Risikofaktor für die psychische Gesundheit darstellt [34, 35]. Aus Präventionssicht empfiehlt sich hier der Einsatz eines Screeningverfahrens zur Identifizierung Betroffener und die zeitnahe Vermittlung an eine Schuldnerberatung.

Der deutliche Zusammenhang zwischen Depression und Einsamkeit bei den ALPE steht in Übereinstimmung mit der Literatur, die sowohl Effekte von Einsamkeit auf Depression als auch von Depression auf Einsamkeit zeigt [4, 36]. Dies legt nahe, dass nicht nur Einsamkeit einen Risikofaktor für (die Schwere von) Depression darstellt, sondern auch umgekehrt und dass beide sich gegenseitig verstärken. Dazu passt, dass unsere Studienteilnehmer*innen einerseits mit 13,8 einen deutlich höheren Depressionswert aufweisen als die Kohorte der umfangreichen, repräsentativen, deutschen NAKO-Studie mit 3,9 [37] und andererseits einen ca. 3‑mal so hohen Anteil an einsamen Personen aufweisen, als dies z. B. in der English Longitudinal Study of Ageing der Fall ist [38]. Die Ergebnisse betonen die Wichtigkeit der Diagnose und Behandlung depressiver Erkrankungen in der Gruppe der ALPE. Hierbei ist eine aktive, aufsuchende Form der Prävention indiziert, da arbeitslose Personen mit psychischen Problemen oftmals eine geringe Bereitschaft aufweisen, sich eigenständig Hilfe im Gesundheitssystem zu suchen, z. B. aufgrund wahrgenommener Stigmatisierung oder falscher Vorstellungen bzgl. einer Behandlung mit Psychopharmaka [39].

Auch bei der Angst zeigt sich ein signifikanter Zusammenhang mit Einsamkeit, welcher sich ebenso in der Literatur wiederfindet und als bidirektional interpretiert werden kann [40–42]. Mit einem Angstscore von 7,3 liegen die ALPE unserer Befragung auch hier wieder deutlich über den Werten eines repräsentativen deutschen Samples (1,4; [43]). Ein hoher Wert ist nicht zuletzt auch deshalb problematisch, weil Angst oft mit pathologischen Verhaltensweisen einhergeht, z. B. im Bereich des Essens oder der Technologienutzung [44, 45]. Geeignete Präventionsansätze könnten Psychoedukation, Entspannung und kognitive Techniken enthalten [46], möglicherweise auch in der Form leicht zugänglicher digitaler Angebote, z. B. über Social Media [47]. Zudem legen unsere Ergebnisse, ähnlich wie bei der Depression, einen hohen Bedarf an psychologischem Screening/Diagnostik nahe.

Anders als in der Literatur [48, 49] findet sich in unserer Stichprobe kein Zusammenhang zwischen Somatisierung und Einsamkeit. Eine potenzielle Erklärung wäre hier, dass möglicherweise existierende Zusammenhänge durch sozial förderliche Effekte der Somatisierung überlagert werden, beispielsweise durch vermehrte Besuche in ärztlichen Praxen, das Aufsuchen von Selbsthilfeforen im Internet oder den Austausch über Symptome. Sicherlich spielt auch eine geringere Stigmatisierungserfahrung im Vergleich zu psychischen Erkrankungen eine Rolle. Hier ist die zukünftige Forschung gefragt, Ursachen zu identifizieren und Zusammenhänge zu quantifizieren. Trotz des fehlenden Zusammenhangs ist es wichtig zu betonen, dass die von uns untersuchten ALPE auch im Bereich der Somatisierung mit 5,6 einen deutlich höheren Durchschnittswert aufweisen als eine repräsentative deutsche Stichprobe (1,5; [43]). Insbesondere die Tatsache, dass es vordergründig um körperliche Symptome geht, erschwert das Erkennen und Behandeln von Somatisierung und impliziert auch die Abklärung körperlicher Erkrankungen, welche in der Gruppe der Arbeitslosen überdurchschnittlich häufig auftreten [50].

Während unsere Studie viele Vorzüge aufweist, wie die Untersuchung einer oft vernachlässigten und schwer erreichbaren Bevölkerungsgruppe, bestehen dennoch Verbesserungsmöglichkeiten für zukünftige Forschung. So wären die Nutzung eines Längsschnittdesigns zum besseren Verständnis kausaler Zusammenhänge und der Einschluss weiterer Schutz- und Risikofaktoren, wie körperliche Erkrankungen und digitale Ressourcen, wünschenswert.

## Fazit

Die Gruppe der ALPE weist eine hohe Belastung sowohl durch psychische Probleme als auch durch Einsamkeit auf. Durch das Aufzeigen von Zusammenhängen zwischen sozialem Netzwerk, Depression, Angst und Einsamkeit sowie deren potenziellen Interaktionen trägt unsere Studie zur Identifikation zentraler Präventionsbereiche bei. Insbesondere Jobcenter bieten sich hier als ideales Setting für die Prävention an.
